# Atomic structure of β″ precipitates in high-Si containing Al–Si–Mg alloy

**DOI:** 10.1186/s42649-026-00128-8

**Published:** 2026-03-21

**Authors:** Saif Haider Kayani, Sang-Ik Lee, Yoon-Ho Lee, Jung-Moo Lee, Kwangjun Euh, Young-Hee Cho

**Affiliations:** 1 Mobility Metals Research Center, Korea Institute of Materials Science, Changwon, 51508 Republic of Korea; 2 School of Materials Science and Engineering, Herbert Gleiter Institute of Nanoscience, Nanjing University of Science and Technology, Nanjing, 210094 China

**Keywords:** Al–Si–Mg alloy, Precipitation, β″-eye, TEM, HAADF, LAADF, Age-hardening

## Abstract

The strength of precipitation-hardened Al–Si–Mg alloys primarily derives from the nanoscale coherent β″ precipitates, which are the dominant contributors to strengthening under peak-aged conditions. Observing these nanoscale precipitates via TEM is particularly challenging due to their strict orientation relationship with the matrix and the subtle atomic number differences between Al, Si, and Mg. In this study, we investigate the precipitation behavior of a high-Si Al–Si–Mg alloy through a comprehensive and systematic microstructural analysis. We demonstrate that the key strengthening mechanism in the peak-aged alloy is the high number density of nanoscale β″ precipitates. The atomic structure of the β″ phase is revealed using HR-TEM, HAADF, and LAADF imaging along the < 010 > Al zone axis. HR-TEM provides clear reflections in the FFT, facilitating effective observation and identification of the β″ phase. Additionally, LAADF imaging proves particularly useful in resolving the atomic structure of the β″ phase. These precipitates display an eye-like morphology, with four Mg and Si atoms arranged in a rim and a central Mg/Al atom, forming a long-range ordered structure. This detailed characterization offers valuable insights into the contribution of β″ precipitates to the alloy’s age-hardening behavior.

**Supplementary Information:**

The online version contains supplementary material available at 10.1186/s42649-026-00128-8.

## Introduction

Al–Si–Mg alloys are a crucial class of lightweight materials known for their high strength and ductility, excellent corrosion resistance, and superior castability (Trink et al. 2025; Raabe et al. 2022; Lee et al. 2024a). These properties make them widely utilized in automotive applications, particularly in the form of complex and intricate components produced through die-casting. These alloys are often used in their as-cast state, where the manufacturing process requires high castability, typically achieved by increasing the Si content to the upper end of the eutectic composition, around 12.6 wt. %. This elevated Si content enhances fluidity during casting (Futas et al. 2018). After casting, the alloys undergo age-hardening treatments, either directly (T5 aging) or following a high-temperature solution treatment (T6 aging). These aging processes lead to the formation of nano-sized metastable precipitates (Bartawi et al. 2023), which significantly contribute to the alloy’s strength and final mechanical properties.

**Fig. 1:**
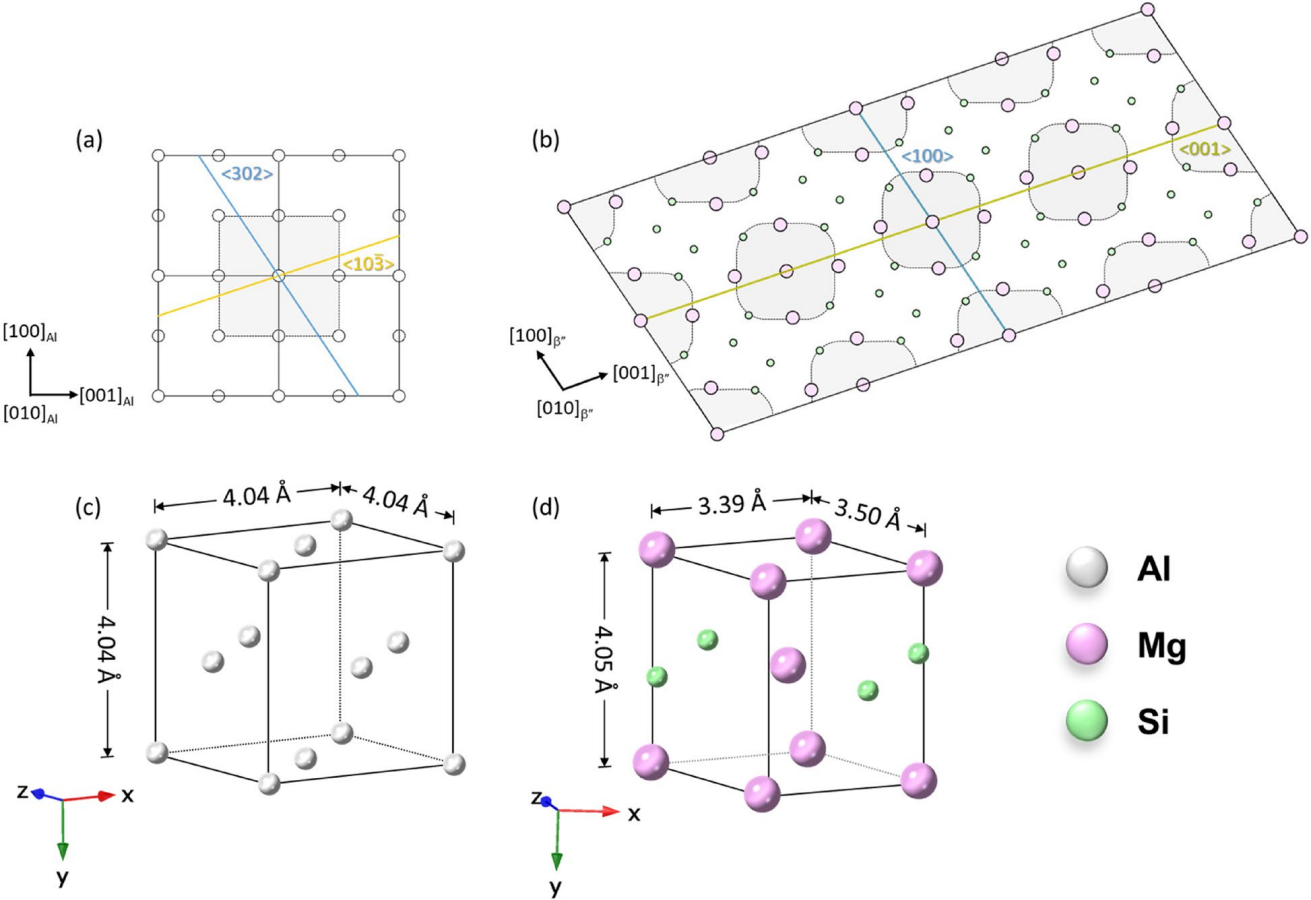
**a** The Al lattice and **b** the β″ lattice along the [010]_Al_//[010]_β″_ direction. The dotted grey squircle represents the β″-eye structure. **c** the Al lattice and **d** the β″-eye unit cells display similar periodicity. The unit cells and lattice models were generated using CrystalMaker® version 10.8.1

In Al–Si–Mg alloys with high Si content, the typical precipitation sequence is as follows: supersaturated solid solution (SSSS) → solute rich cluster → GP zones → β″ → β′, U1, U2, B′ → β and Si precipitates (Edwards et al. 1998; Marioara et al. 2005; Cui et al. 2023). The principal strengthening phase in these alloys is the β″ phase, which adopts a needle-like morphology and possesses a monoclinic unit cell with parameters a = 1.516 nm, b = 0.405 nm, c = 0.674 nm, and β = 105.3°, with a space group of C2/m (Andersen et al. 1998; Chen et al. 2006). The β″ unit cell contains two structural units referred to as the β″-eye or low-density cylinders (LDC) (Huis et al. 2007). The β″-eye structure is analogous to an FCC Al unit cell, with Mg atoms occupying the four corner positions and Si atoms located at the four face centers, while the central column consists of either Mg/Al atoms (Andersen et al. 2018; Hasting et al. 2009). Due to the larger size of these central atoms, they induce distortion in the surrounding lattice. To minimize this distortion, the larger Mg/Al atoms shift by half a unit cell along the b-axis, moving into an interstitial position between four smaller Si atoms. This shift results in a more symmetric and spacious configuration, thereby reducing the elastic strain within the β″ structure (Wenner et al. 2017; Ninive et al. 2014). When observed in 2D images using TEM, the β″-eye appears as a central atom surrounded by four Mg and Si atoms in nearly a circular arrangement, resembling an “eye” structure, as illustrated in Fig. 1b. This unique morphology and structural configuration contribute significantly to the strengthening mechanisms of Al–Si–Mg alloys.

The orientation relationship between β″ and the Al matrix is identified as: [100]_β″_//[302]_Al_, [010]_β″_//[010]_Al_, [001]_β″_//[10 $\overline{3 }$]_Al_ (Andersen et al. 2005). The [010]_β″_ direction represents the long axis of β″ needles, which exhibit a periodicity of 4.05 nm, similar to the structure of FCC Al, as shown in Fig. 1c, d. Along this axis, β″ precipitates are fully coherent with the Al matrix, while they are semi-coherent in the other two directions. Due to this strict orientation relationship, the observation of β″ precipitates via transmission electron microscopy (TEM) is highly dependent on the zone axis (Andersen et al. 1998). Specifically, when the TEM zone axis is aligned with < 001 > _Al_, the (010)_β″_ plane is nearly parallel to {010}_Al_, and the needle axis of β″ aligns with < 10 $\overline{3 }$>_Al_ (Marioara et al. 2024; Li et al. 2024). In this orientation, Al atoms project as a simple square FCC grid, while the β″ Mg/Si sublattice produces an additional periodic modulation that does not coincide with the Al columns. This results in diffraction extra β″ reflections and streaks that are clearly separated from the fundamental Al spots, allowing for easy phase identification and structural refinement. However, when the TEM zone axis is aligned with < 110 > _Al_ or < 111 > _Al_, the β″ atomic columns overlap with Al columns, causing the additional β″ reflections to lie close to the matrix reflections. In this case, the nanoscale precipitates become difficult to distinguish or are essentially invisible against the Al background.

The application of aberration-corrected high-angle annular dark-field (HAADF) imaging to study the atomic structure of β″ is limited by the small difference in atomic numbers between Mg (Z = 12), Al (Z = 13), and Si (Z = 14). This results in insufficient Z-contrast to clearly identify the atomic columns in the nm-sized clusters embedded within the thicker Al matrix (Liu et al. 2021). HAADF imaging typically employs relatively large detector collection angles, which reduces the detected signal and, consequently, the signal-to-noise ratio (Hell et al. 2024). In this mode, the noise can be mitigated by increasing the incident beam voltage, increasing the dwell time, and using very thin TEM foils with minimal local bending. For commercial high-Si Al–Si–Mg cast alloys, TEM specimen preparation is therefore often restricted to focused ion beam (FIB) lift-out, because electro-polishing readily produces Si-particle-associated pitting (Shankar et al. 2003). However, FIB milling can introduce lamella bending as well as ion-beam-induced damage, which further complicates HAADF imaging in these alloys. In contrast, most prior studies on wrought 6xxx-series alloys with comparatively low Si/Mg ratios have employed electro-polishing to avoid FIB-induced artefacts (Marioara et al. 2024; Li et al. 2024; Liu et al. 2021) and have not encountered these challenges.

Therefore, to address these challenges, we present an efficient method to visualize the atomic structure of β″ in high-Si Al–Si–Mg alloys using low-angle annular dark-field (LAADF) imaging, which provides contrast from the small atomic displacements rather than relying on Z-contrast. We investigated the age-hardening behavior and precipitation microstructure of a peak-aged Al-10.2Si-0.35Mg alloy through correlative microstructural analysis. The atomic structure of the eye-like features was examined in relation to the primary hardening phase, β″, to clarify their contribution to peak hardness.

## Experimental

**Fig. 2:**
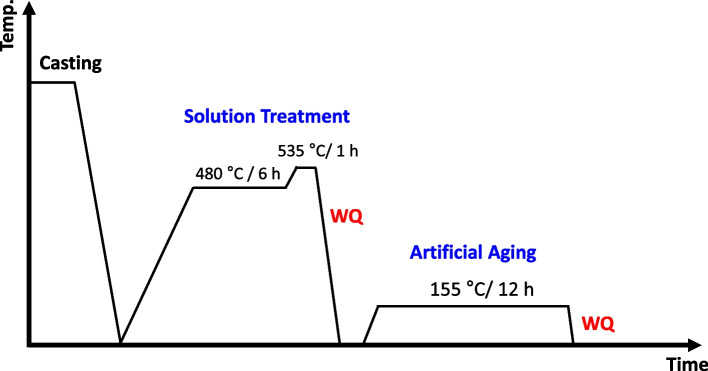
Schematic representation of the heat treatment process used in this study. WQ denotes water quenching

The Al–Si–Mg alloy was produced through conventional gravity casting. The detailed alloy fabrication procedure is described in Kayani et al. (2024). The composition of the as-cast alloy, presented in Table 1, was analyzed using inductively coupled plasma optical emission spectroscopy (ICP-OES; Thermo Scientific iCAP 6500). The as-cast alloy underwent a solution treatment at 480 °C for 6 h, followed by eutectic Si spheroidization at 535 °C for 1 h. Afterward, the alloy was aged directly at 155 °C without any natural aging at room temperature. A schematic representation of the heat treatment process is shown in Fig. 2. For microstructure analysis, samples were prepared using conventional grinding and polishing methods. Optical imaging of the polished surfaces was performed using an optical microscope (OM; Nikon, MA200). Secondary phases were examined using a field-emission scanning electron microscope (FE-SEM; JEOL JSM-7900F) equipped with an Oxford energy-dispersive X-ray spectroscopy (EDS) detector. Vickers hardness testing was conducted to assess age-hardening behavior using a Mitutoyo Co. HM200 hardness tester. A load of 0.01 kgf with a dwell time of 15 s was applied for each hardness measurement.

**Table 1:** The nominal composition of the Al–Si–Mg alloy in the present study

**Alloy/Composition**	Si	Mg	Zr	Fe	Sr	Al
Al–Si–Mg (wt. %)	10.2	0.35	0.1	0.2	0.02	Bal

**Fig. 3:**
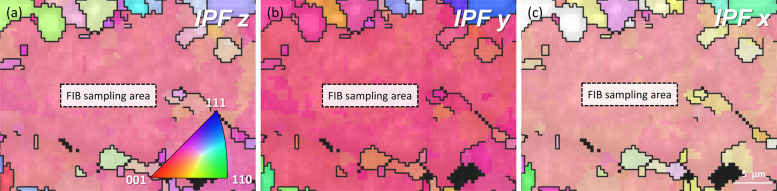
Inverse pole figures of an Al–Si–Mg alloy along the **a** z, **b** y, and **c** x axes. The reddish color observed in all three axes indicates that the grain orientation is predominantly aligned with the < 100 > direction across all orientations

TEM samples were prepared using a FIB on a Thermo Fisher Helios G5 workstation. Previous studies predominantly employed electro-polishing as a preparation technique since it prevents surface amorphization and minimizes damage to the atomic structure of precipitates. In contrast, ion bombardment can knock atoms out of the lattice, potentially altering the structure of the precipitates. To mitigate this effect, ion milling was performed at low current (~ 50 pA) and voltage (5–3 kV) following pre-thinning. The final thickness of the TEM samples was approximately 80 nm. Another challenge associated with FIB preparation is the limited analysis area (3 × 6.5 µm) available for imaging, compared to the larger 3 mm discs obtained by electro-polishing. Due to the small sample area, FIB lamellae often require significant tilting to align with a desired zone axis, especially when the sample orientation is unknown. To ensure that TEM images were captured along the < 001 > _Al_ zone axis, electron backscatter diffraction (EBSD) analysis was conducted on the sample prior to FIB thinning, using a FE-SEM. Figure 3a-c display the inverse pole figure (IPF) maps of the FIB sample. A single grain with an orientation close to < 001 > Al in the IPF x, y, and z axes was selected. This approach ensured a nearly perfect cube-cube relationship between the sample and grain (crystal), as illustrated in Fig. S1(a, b). Consequently, the lamella’s z-plane, aligned with the beam, and the x and y planes, normal to the beam, were all oriented along < 001 > _Al_, minimizing the tilt required to achieve the < 001 > _Al_ zone axis during the experiment.

TEM imaging was performed using both HAADF and LAADF modes on a Thermo Fisher Themis Z operated at 300 keV. The probe size and convergence angle were set to 0.1 nm and 17.9 mrad, respectively. The annular dark-field (ADF) detectors enabled the simultaneous collection of both LAADF and HAADF signals. LAADF imaging was performed with inner and outer collection angles of 20 mrad and 78 mrad, respectively, while HAADF imaging used inner and outer collection angles of 83 mrad and 203 mrad, respectively. The raw HAADF and LAADF images are presented without any image filtration.

## Results

### Microstructure analysis

**Fig. 4:**
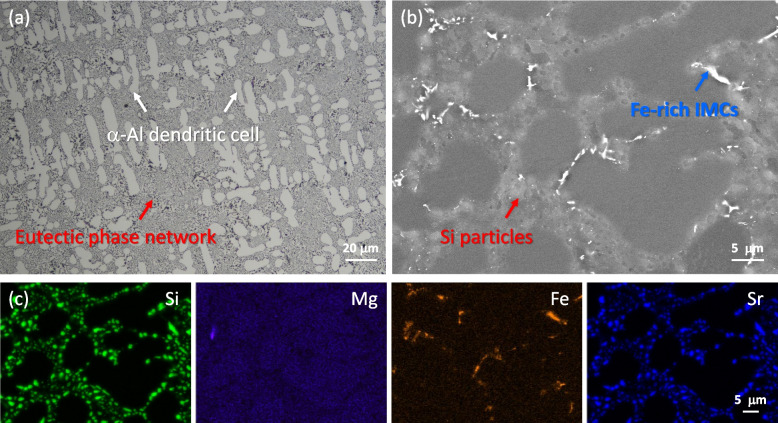
**a** OM and **b** BSE SEM images, and the corresponding EDS elemental maps of Al–Si–Mg alloys in the solution-treated and aged condition

Figure 4a presents an OM image of the sample following solution treatment, showing the formation of α-Al dendritic cells (indicated by white arrows), which are surrounded by a network of eutectic phases (red arrows). Due to the two-step solution treatment, the eutectic phase particles exhibit a disintegrated morphology within the network, indicating their spheroidization during second stage solution treatment at 535 °C. The backscattered electron (BSE) SEM image and the corresponding energy dispersive spectroscopy (EDS) analysis in Fig. 3b, c reveal that the bright contrast particles are composed of an Fe-rich intermetallic phase, while the light-grey contrast particles correspond to eutectic Si.

### Age-hardening behavior

**Fig. 5:**
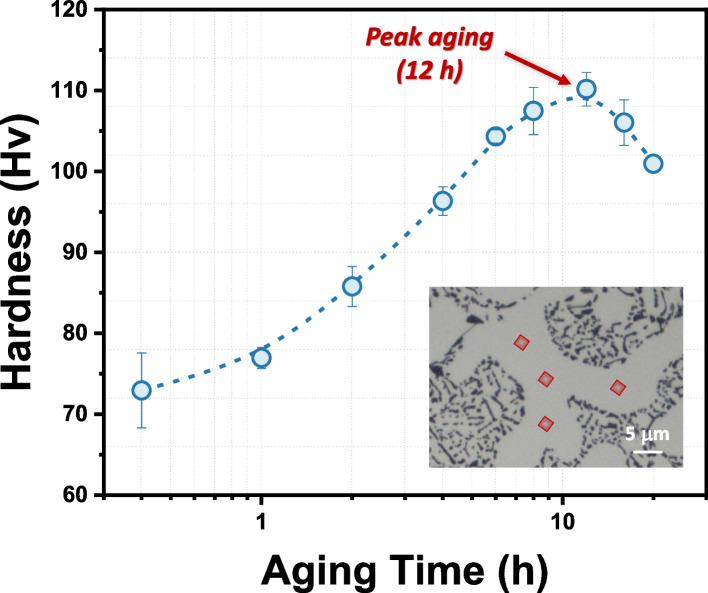
Age-hardening curve of the Al–Si–Mg alloy at 155 °C following solution treatment. The inset displays the region within the α-Al dendrite selected for indentation testing

Figure 5 illustrates the age-hardening behavior of the alloy during artificial aging at 155 °C. Initially, the alloy exhibits a Vickers hardness of 72.94 Hv following solution treatment, which increases steadily over time. After 12 h of aging, the hardness reaches its maximum value, peaking at 110.18 Hv, corresponding to the peak-aging condition due to the high number density of coherent β″ precipitates. However, with further aging beyond 12 h, the hardness begins to decrease as the coherent β″ precipitates transition into semi-coherent precipitates, resulting in reduced hardness.

### Precipitation analysis

**Fig. 6:**
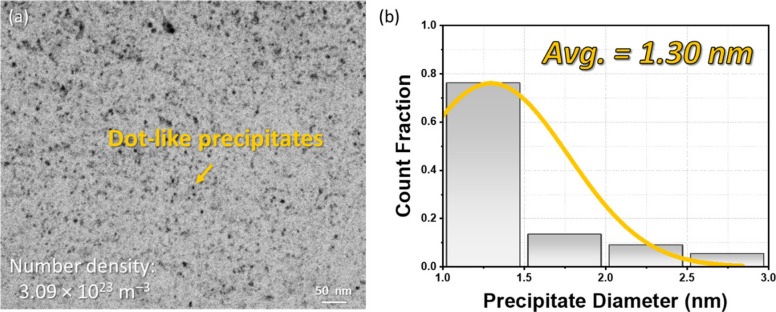
**a** BF TEM image and **b** distribution of average precipitate diameters in a peak-aged Al–Si–Mg alloy

Figure 6a presents the bright-field (BF) TEM image of the Al–Si–Mg alloy peak aged at 155 °C following two-step solution treatment. Several dot-like precipitates are visible, corresponding to the cross-sectional view of the β″ phase. The average diameter and number density of the precipitates were calculated to be 1.30 nm and 3.09 × 10^–4^ nm^–3^, respectively (Fig. 6b).

**Fig. 7:**
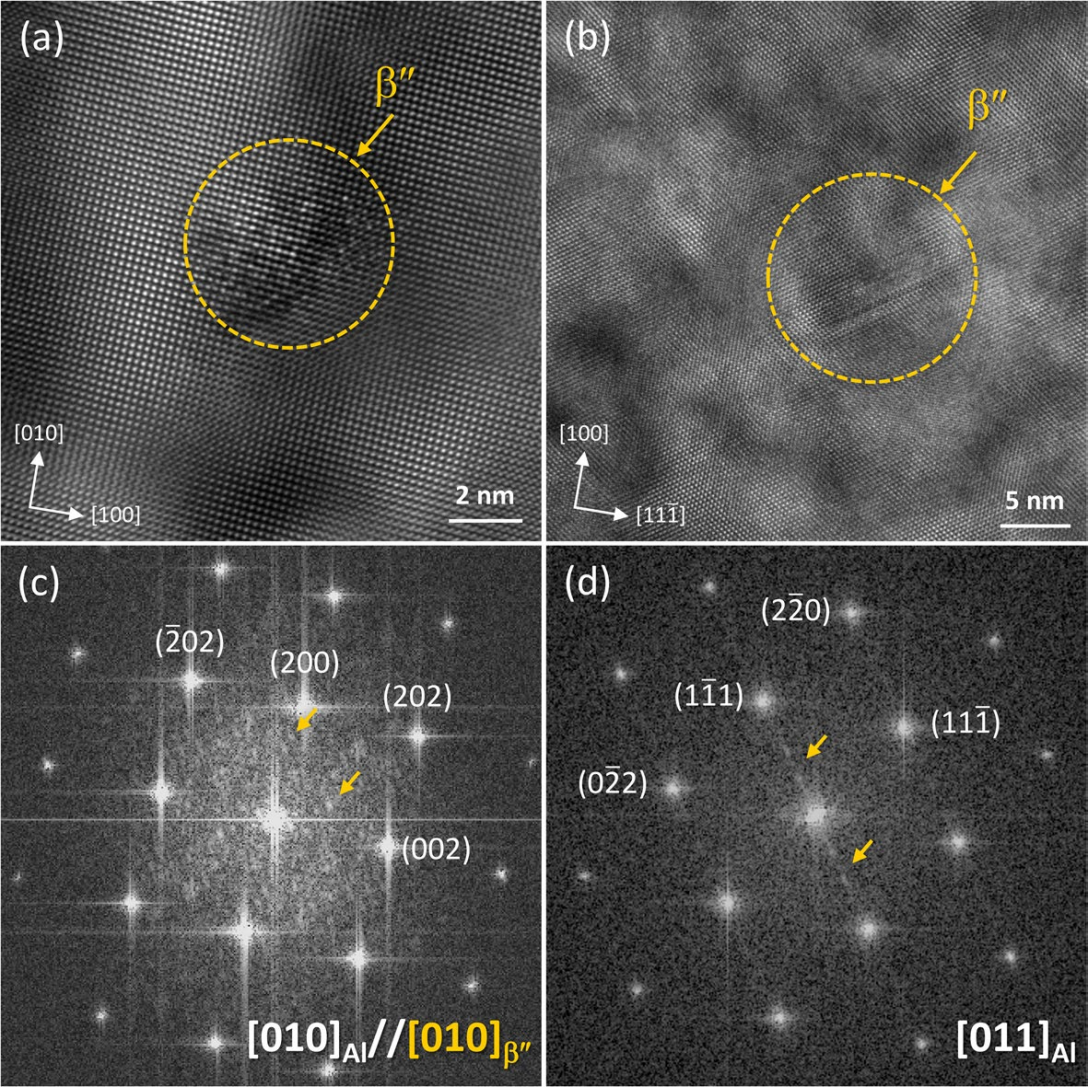
**a**, **b** HR-TEM images and **c**, **d** corresponding FFTs of the Al–Si–Mg alloy along the **a**, **c** [010]_Al_ and **b**, **d** [011]_Al_ zone axes

Figure 7a, b shows the high-resolution TEM (HR-TEM) images of β″ recorded along the [010]_Al_ and [110]_Al_ zone axes. Compared to the Al matrix planes, dot-like of β″ phase is observed along the [010]_Al_ zone axis, as indicated by the dashed yellow circle. The corresponding fast Fourier transform (FFT) in Fig. 7(c) reveals distinct β″ reflections. It is noteworthy that this configuration corresponds to a cross-section of the β″ precipitates, with some β″ planes nearly parallel to the Al planes, as highlighted by the yellow arrows in Fig. 7d. On the other hand, a streak-like morphology is observed along the [110]_Al_ zone axis, and only streaks are seen along the {111}_Al_ planes in the FFT (Fig. 7d). In this case, multiple β″ atomic columns project on top of Al columns, and the additional β″ reflections are close to the strong matrix reflections. As a result, the precipitates become difficult to distinguish, and only streaks are visible, which may correspond to precipitates that are essentially invisible against the Al background.

### β″-eye observation via HAADF and LAADF imaging

**Fig. 8:**
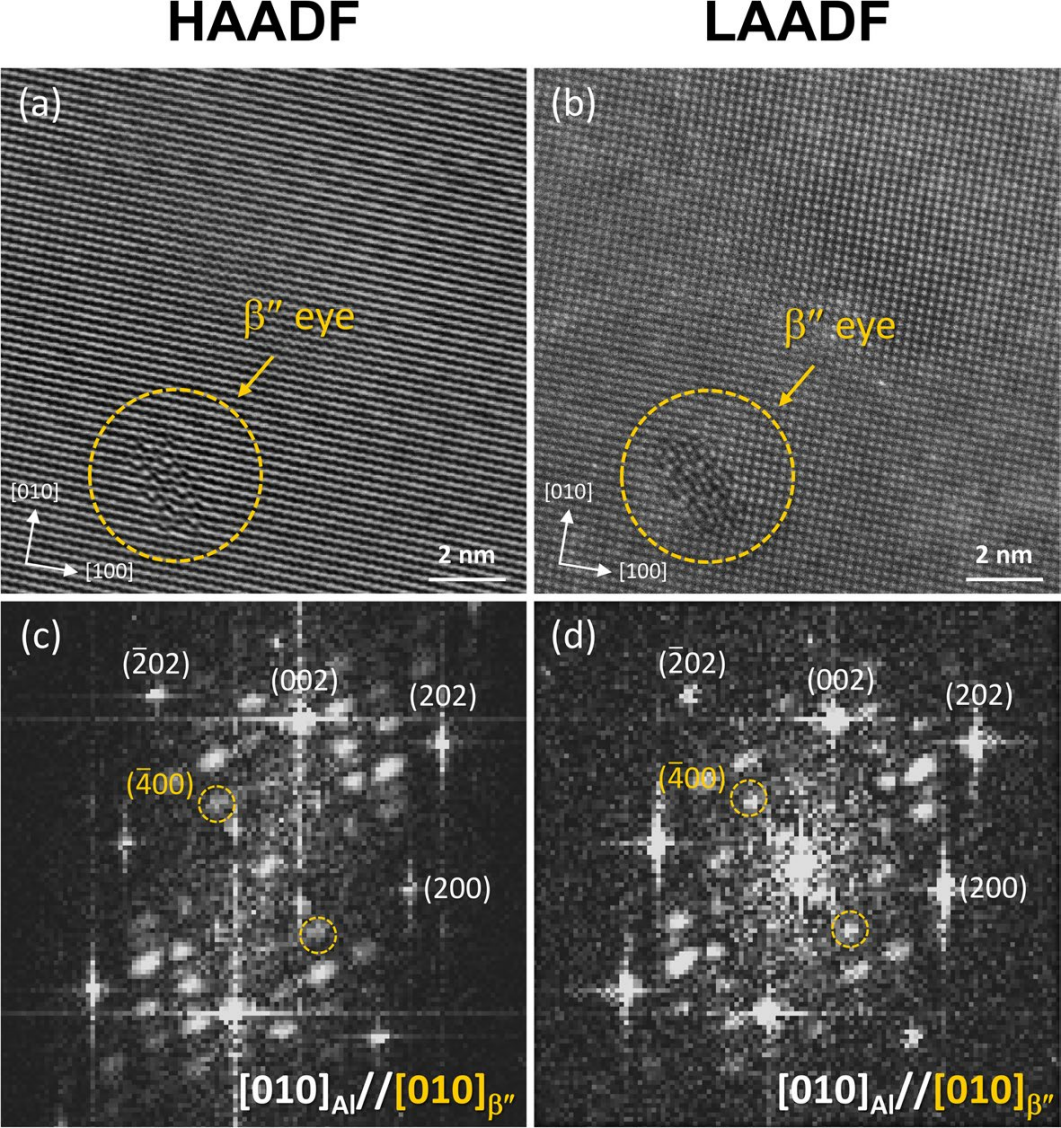
**a** HAADF and **b** LAADF images, along with **c** and **d** their corresponding FFTs, showing the β″ atomic structure in an Al–Si–Mg alloy along the [010]_Al_ zone axis

Figure 8a, b presents HAADF and LAADF images of a peak-aged Al–Si–Mg alloy, showing β″ precipitates along the [010]_Al_ zone axis. The corresponding FFTs for both images are shown in Fig. 8c, d. In both HAADF and LAADF images, the atomic planes of the β″ precipitates are distinctly different from that of the surrounding matrix, as highlighted by the dashed yellow circles. However, due to the high-angle scattering in HAADF, the contrast of the β″ precipitates is significantly enhanced, making it difficult to distinguish between Si, Mg, and Al atoms within the precipitates. In contrast, the low-angle scattering in LAADF provides improved atomic contrast, allowing for a clearer distinction between Mg and Si atoms within the precipitates. This enhanced contrast reveals the ordered structure of the β″ precipitates, which exhibits an eye-like feature. The structure consists of four larger Mg atoms and smaller Si atoms forming the rim, with a single larger atom positioned at the center of the eye.

## Discussions

The high strength of Al alloys arises from several strengthening mechanisms, including solid solution, eutectic and intermetallic particles, precipitation, grain boundary, and dislocation strengthening (Park et al. 2021; Ma et al. 2014; Lagerpusch et al. 2001; Suwanpreecha et al. 2019; De Luca et al. 2019). Al–Si–Mg alloys, which are typically used in their as-cast condition in service applications, do not benefit from strengthening processes such as rolling and extrusion (minimal grain boundary and dislocation strengthening). In wrought Al–Si–Mg alloys, peak-aged strengthening is governed primarily by the high number density of matrix precipitates, and quantitative precipitate–property relationships are well established under conditions where the matrix chemistry and defect structure are comparatively uniform (Saito et al. 2018). In high-Si Al–Si–Mg cast alloys, precipitation evolves within α-Al dendrites surrounded by eutectic Si and Fe-rich intermetallic particles, which can perturb precipitation through coupled solute–vacancy–strain effects in the matrix (Marioara et al. 2005; Ding et al. 2022). For example, the high number density of eutectic Si and Fe-rich particles redistributes solute near cell boundaries, promoting spatially heterogeneous precipitation during cooling and subsequently modifying precipitation kinetics during aging (Kayani et al. 2024). Thermal-mismatch strains between these rigid constituents and the α-Al matrix further redistribute vacancies and dislocations, thereby altering β″ nucleation and growth locally (Wang et al. 2022). Nevertheless, once the α-Al matrix is adequately supersaturated, β″ remains the dominant precipitation-strengthening phase at peak aging in cast Al–Si–Mg alloys (Lee et al. 2024b). The eutectic particles primarily facilitate crack initiation and provide preferential propagation paths during deformation, while β″ act as the principal source of strengthening within Al-matrix dendrites (Kayani et al. 2025). At the nanoscale, coherent β″ precipitates impede dislocation glide and are predominantly sheared during deformation.

Due to their high number density in the peak-aged condition, precipitation shearing is the dominant mechanism responsible for the high yield strength of the alloy, as reported in various studies (Kayani et al. 2025; Yang et al. 2021). However, with prolonged aging, the β″ precipitates coarsen and transform into semi-coherent precipitates, resulting in a reduction in precipitate number density. When interacting with dislocations during deformation, dislocations are unable to shear these larger precipitates and instead bypass them through Orowan looping (Kayani et al. 2025; Teichmann et al. 2013; Wang et al. 2021). This mechanism becomes the dominant strengthening process in alloys subjected to prolong aging treatments, as indicated by hardness reduction in Fig. 5. Therefore, we conclude that the high number density of coherent β″ precipitates is the primary strengthening phase in the peak-aged, high-Si Al–Si–Mg alloy studied in the current research.

Furthermore, this study demonstrates a systematic experimental approach to resolve the atomic structure of coherent β″ precipitates in Al–Si–Mg alloys. A critical aspect of this approach is the selection of the < 010 > _Al_ zone axis, which is key to successfully imaging the β″-eye structure (Andersen et al. 1998). To achieve this, it is effective to prepare a TEM lamella sample with the IPF x, y, and z directions aligned parallel to the < 010 > _Al_ zone axis of the crystal. When the TEM sample is not aligned with this zone axis, significant tilting is often necessary to bring the sample’s zone axis into alignment with < 010 > _Al_. However, this tilting can lead to poor diffraction conditions for observing nanometer-sized precipitates, due to the change in the sample’s effective thickness (t_e_). As shown in Fig. S2, increasing the tilt angle during TEM increases t_e_. In the case of precipitates that are only a few nanometers in size, diffraction from these precipitates can still be observed, even under high tilt conditions. However, this alignment is ideal for observing the β″-eye structure, as the diffraction patterns from the β″ precipitates may overlap with the strong matrix diffraction, complicating the observation.

**Fig. 9:**
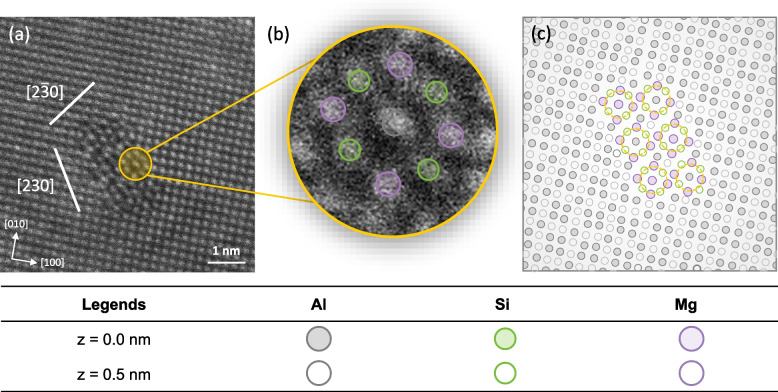
**a** High-magnification LAADF image of the β″-eye structure in an Al–Si–Mg alloy along the [010]_Al_ zone axis. **b** Atom positions of Al, Mg and Si within the β″-eye structure. **c** Schematic representation of the six β″-eye structures observed in (**a**)

Based on our experimental and analytical approach, we clearly identified the atomic structure of β″ precipitates, consistent with past reports (Marioara et al. 2024; Li et al. 2024; Liu et al. 2021). The LAADF image reveals a well-defined ordering of Mg and Si atoms, showcasing distinct motifs within the β″ precipitates. Previous studies have also reported β″-eye features in GP zones, which are considered the precursor phase to β″. However, the multi-eyed structures observed in GP zones are distinguishable from β″ by the number of eyes in the precipitate’s cross-section (Marioara et al. 2024). For instance, it has been reported that at least six eyes are required to form the long-range ordered monoclinic structure of β″ in three dimensions (Andersen et al. 2018; Li et al. 2024). To further illustrate this, we present a high-magnification LAADF image of β″ in Fig. 9a, with a single β″ eye depicted in Fig. 9b, where Si and Mg atoms are highlighted in green and purple circles, respectively. The schematic in Fig. 9c demonstrates that the precipitate possesses six eyes, as indicated by the dashed yellow circles, confirming its long-range ordered β″ structure. While previous literature has documented β″ precipitates with more than six eyes, these are often not observed to display the perfect monoclinic unit cell of β″. Such precipitates are typically classified as disordered β″ phases (Hell et al. 2023; Mørtsell et al. 2017). These disordered structures tend to exhibit a mixture of stacking variations. In contrast, our observations show that the β″ precipitates in this study maintain a fully consistent ordering, without the presence of these stacking irregularities, even under peak-aged conditions.

## Conclusion

This study investigates the age-hardening and precipitation behavior of a high-Si containing Al–Si–Mg alloy. The alloy achieved peak hardness after 12 h of aging at 155 °C. Through a combination of microstructural analyses, we demonstrate that the primary contributor to the alloy’s hardening is the high density of nanoscale β″ precipitates. The atomic structure of the β″ phase is elucidated using HR-TEM, HAADF, and LAADF imaging along the < 010 > _Al_ zone axis. HR-TEM analysis reveals distinct reflections in the FFT, allowing for efficient observation and identification of the β″ phase along the < 010 > _Al_ zone axis. While, the low scattering angle in LAADF imaging proves to be an effective method for resolving the atomic structure of the β″ phase. The β″ structure is characterized by an eye-like feature composed of four Mg and Si atoms forming the rim, with one central Mg/Al atom in the middle. The β″ precipitates consist of six such eyes, forming a long-range ordered structure.

## Supplementary Information

Supplementary Material 1.

## Data Availability

The raw/processed data required to reproduce these findings cannot be shared at this time as the data also form part of an ongoing study.
