# Supplementary material for: Atomic structure of β″ precipitates in high-Si containing Al–Si–Mg alloy
Source: Appl Microsc. 2026 Mar 21;56:8. doi: 10.1186/s42649-026-00128-8 (PMC13005791; doi:10.1186/s42649-026-00128-8)
Supplement: Supplementary file 1 — Supplementary Material 1. [file 42649_2026_128_MOESM1_ESM.docx]

**Atomic structure of β″ precipitates in high-Si containing Al-Si-Mg alloy**

Saif Haider Kayani^1,2,*^, Sang-Ik Lee^2^, Yoon-Ho Lee^2^, Jung-Moo Lee^2^, Kwangjun Euh^2^, Young-Hee Cho^2,*^

^1^School of Materials Science and Engineering, Herbert Gleiter Institute of Nanoscience, Nanjing University of Science and Technology, Nanjing 210094, China

^2^Mobility Metal Research Center, Korea Institute of Materials Science, Changwon, 51508, Republic of Korea

^*^Corresponding Author: [saifkayani@gnu.ac.kr (S.H](mailto:saifkayani@gnu.ac.kr%20(S.H). Kayani), [y.cho@kims.re.kr](mailto:y.cho@kims.re.kr) (Y. H. Cho)

**Supplementary Materials**

**
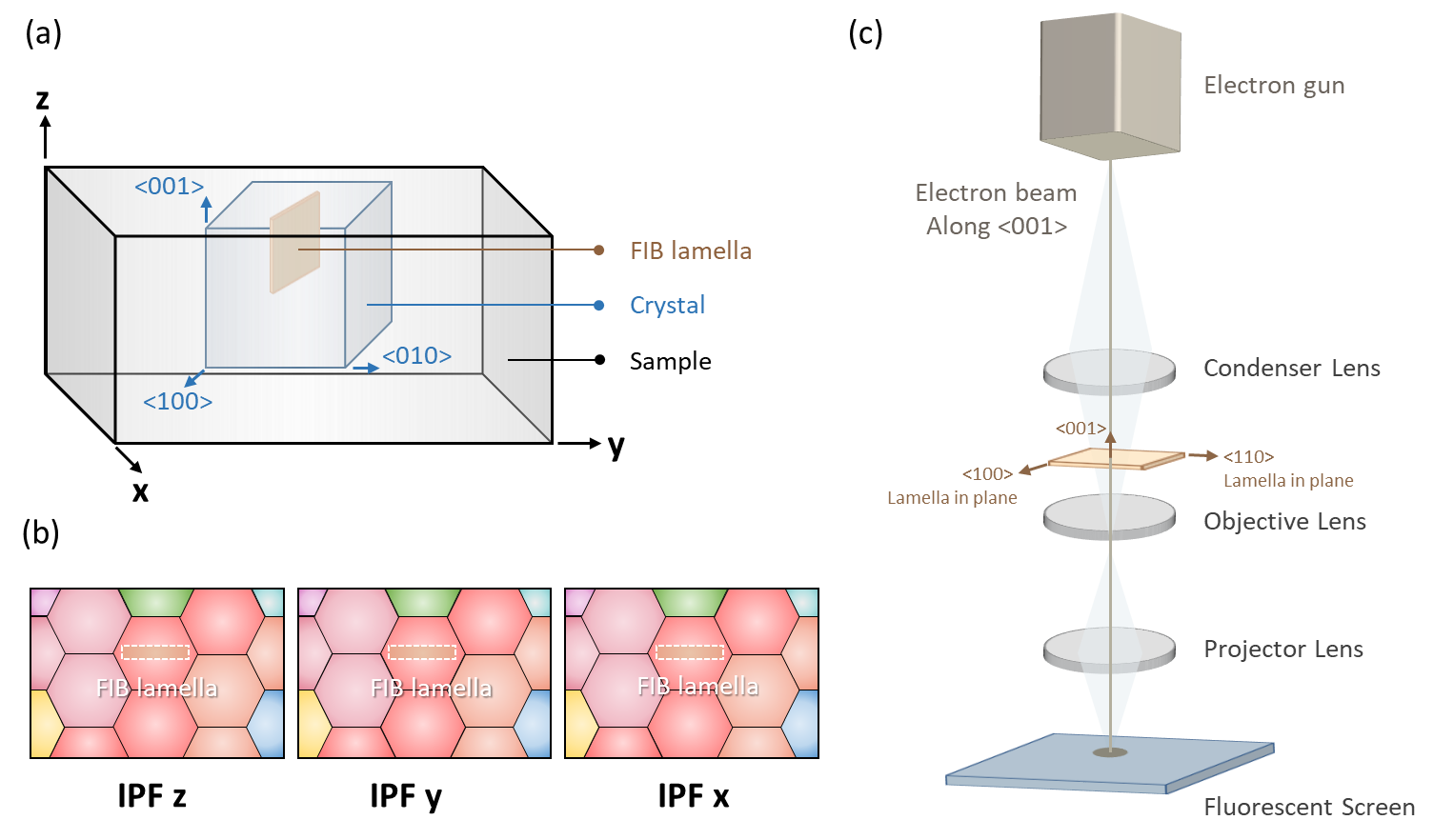
**

**Fig. S1.** (a) Schematic illustrating the cube-cube relationship between sample and crystal (grain). (b) IPF maps showing the orientations along the z, y, and x axes. (c) Schematic of TEM, where the electron beam is aligned with the z-axis of the FIB lamella while passing through the <100> crystallographic direction in the planes.

**
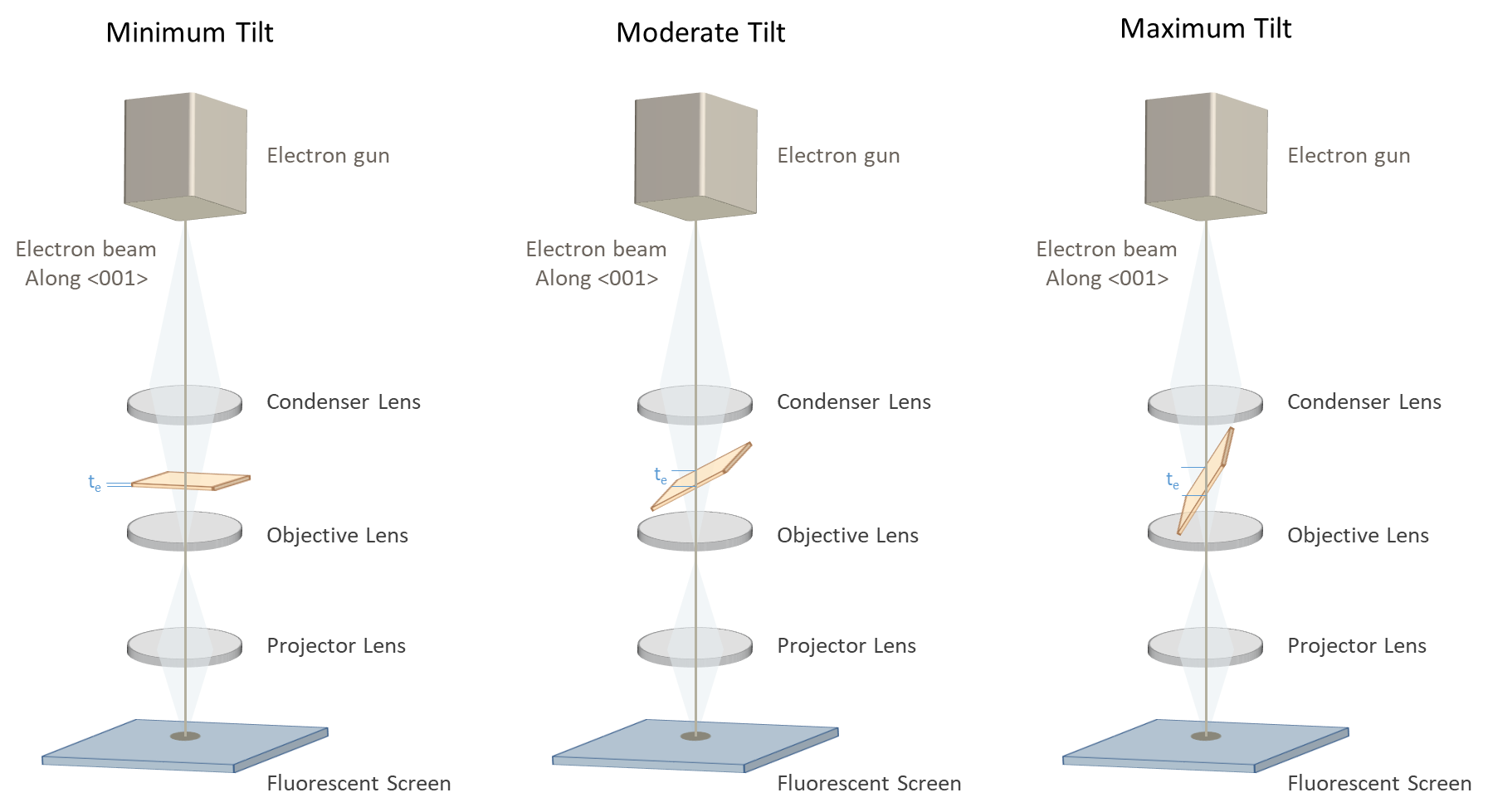
**

**Fig. S2.** Variation in the effective thickness (tₑ) of the sample as a function of tilt during TEM analysis.


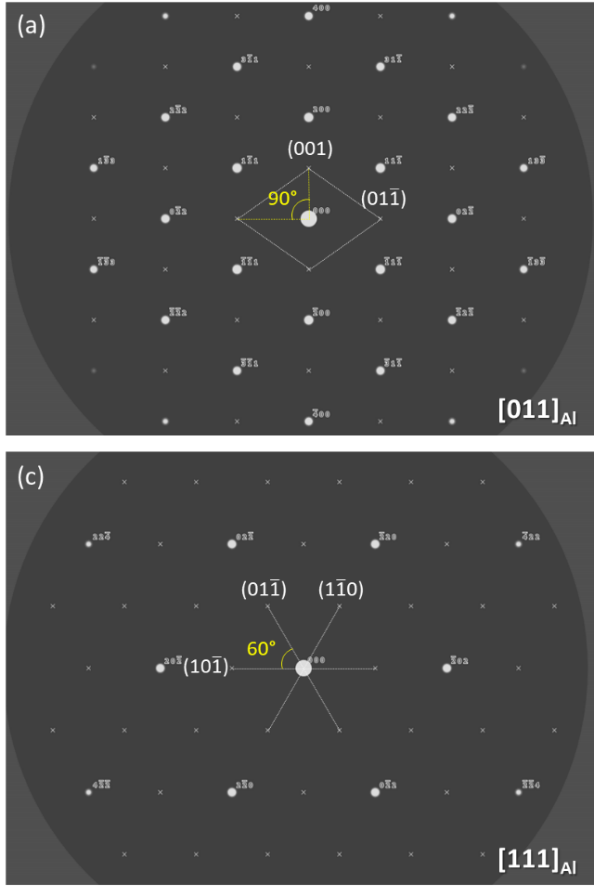


**Fig. S3.** Diffraction patterns of Al along [011] and [111] zone axis simulated using CrystalMaker® version 10.8.1.


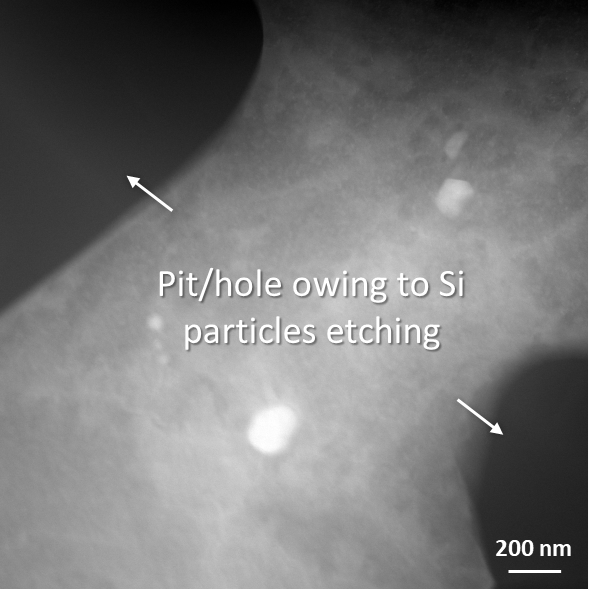


**Fig. S4.** BF-TEM of of the Al-Si-Mg alloy; the TEM sample was prepared using electro-polishing.
